# “I'm sick of being called a hero – I want to get paid like one”: Filipino American frontline workers' health under conditions of COVID-19 and racial capitalism

**DOI:** 10.3389/fpubh.2022.977955

**Published:** 2022-11-23

**Authors:** Cindy C. Sangalang

**Affiliations:** ^1^Department of Social Welfare, Luskin School of Public Affairs, University of California, Los Angeles, Los Angeles, CA, United States; ^2^Asian American Studies Department, Social Sciences Division, University of California, Los Angeles, Los Angeles, CA, United States

**Keywords:** frontline workers, COVID-19, health, racial capitalism, Filipino Americans

## Abstract

Although the era of COVID-19 has reaffirmed the vital role of frontline workers in maintaining a functional society, the ongoing pandemic has taken a devastating toll on their health and well-being. In the United States, Filipino American frontline workers in healthcare and service industries have endured threats to their health, safety, and economic livelihood throughout the pandemic and against the broader backdrop of racialized and xenophobic hate directed toward Asian Americans. Drawing on a qualitative approach, the current study explores work-related health risks and effects of the pandemic for Filipino American frontline workers. Data come from the qualitative arm of a larger mixed-methods study that used a community-based participatory research approach. The current analysis is based on focus group data with thirty-five Filipino American frontline workers, a majority of whom were migrants, that worked across healthcare, caregiving, education, childcare, food services, and retail industries. Situated through the lens of racial capitalism, themes included: (1) work-related stress, tensions, and trauma, (2) anti-Asian racism and intersections with age- and gender-based violence, and (3) working while ill and distressed. Study findings can inform interventions and policies to improve health, occupational environments, and labor conditions in order to support minoritized communities disproportionately affected by COVID-19.

## Introduction

Although the COVID-19 pandemic has re-affirmed the vital role of essential and frontline workers play in maintaining a functioning society, it has also taken a devastating toll on the health and well-being of these workers. Definitions of essential workers vary by region and have evolved over the course of the pandemic, yet they are generally employees within healthcare, food processing and services, agriculture, child care, janitorial work, manufacturing, retail, and other critical sectors. Among essential workers, frontline workers are those who are unable to work from home, and because they provide labor in person, are placed at greater risk of exposure to COVID-19 (1, 2). People of color, migrants, women, and other minoritized groups are disproportionately represented as frontline workers across various sectors (2–5). Among the most marginalized within this labor force, many lack health insurance (6) and face poorer health due to social and economic disadvantages that existed prior to the pandemic (3), contributing to a widening of social, economic, and health inequities as the pandemic lingers.

### Filipino frontline workers in the U.S.

Filipino Americans represent a significant proportion of frontline workers in health and service industries, making them vulnerable to contracting and dying from COVID-19. For example, data from the early stage of the pandemic revealed Filipino Americans accounted for at least 35% of deaths due to COVID-19 among Asian Americans in California despite comprising about 25% of the state's Asian American population (7). Nationally, Filipinos comprise 4% of all registered nurses in the U.S. but constitute 31.5% of all COVID-19-related fatalities, as they are more likely to work the frontlines of intensive and acute care units (8, 9). Beyond those employed in healthcare, many Filipinos are vulnerable to precarious work conditions and trafficking (e.g., home health and domestic care, hospitality, “guest worker” positions), particularly for the hundreds of thousands (310,000+ as of 2017) of undocumented Filipino migrant workers in the U.S. (10). Furthermore, a 2019 AAPI California Worker's Survey indicated that 22% of Filipino Americans were working and also struggling with poverty (11), increasing the likelihood of workers feeling compelled to continue working even if they fall ill. This has all occurred against the broader backdrop of racialized and xenophobic hate directed toward Asian Americans (12), including highly-publicized cases targeting Filipino Americans that have created a climate of fear and anxiety about issues of community safety (13).

The existing body of research on Filipino American frontline workers highlights various social, economic, and health challenges they face. Given the sizeable representation of Filipino Americans in nursing, numerous studies have highlighted heightened risk for COVID-related death (8), as they are more likely than White counterparts to work in acute and long-term inpatient settings (14). In addition, compared to White counterparts, Filipino nurses (RNs) report lack of advancement and burnout as occupational stressors yet are less likely to consider leaving their position (14). Other research with Filipino home-health workers and those employed in nursing homes and assisted living facilities underscores exploitative employment conditions (e.g., irregular or lack of mandated rest and meal breaks), lack of safety protections (e.g., limited personal protective equipment, or PPE), risk of physical injury on the job, and lack of health insurance or limited access to medical care (15) that existed prior to the pandemic. Many of the Filipino workers in healthcare and caregiving are migrants (16), with research showing a positive association between job-related stressors and chronic health conditions (17) as well as a link between job dissatisfaction and psychological distress (18) among Filipino migrants overall. Despite the mounting evidence exploring occupational stressors and their effects on health, few studies have examined the experiences of Filipino American frontline workers employed across diverse industries during the pandemic (3, 19).

### Conceptual framework: Racial capitalism

Racial capitalism offers a framework for understanding how racial and capitalist hierarchies work together to create the structures and conditions that contribute to social and health inequities. Emanating from the contributions of radical African and Black intellectuals, and synthesized, expanded, and elevated by Cedric Robinson (20), racial capitalism posits that racism and capitalism are intertwined, such that capital accumulation among elites is dependent on the labor and exploitation of Black people. As articulated by Bright et al. (21): “The theory of racial capitalism proposes an origin story for how it is that the global economy came to be racially stratified and (in the main) organized along capitalist lines” (p. 1). Given legacies of colonialism and imperialism that have depended on the extraction and exploitation of labor, land, and resources of Indigenous people, enslaved Africans, and other racialized peoples, racial capitalism is a relevant framework across racially minoritized workers (22, 23), and offers a historical lineage and context for understanding the interdependence of racism and class oppression as a fundamental cause of disease (24) and form of structural violence influencing health (25).

Turning to the current public health crisis, McClure et al. (26) argue that racial capitalism is a key driver of racial disparities in COVID-19 infections given the primacy of occupational settings in viral transmission among workers of color in low-wage, service-sector, and healthcare industries. Drawing on analysis of workers' compensation claims, they critique the dominant narratives that focus on individual-level and culture-based risks for COVID-19 (e.g., comorbidities, health literacy, multigenerational household arrangements) that mask the complicity of industries and institutions in failing to protect workers' health and safety. As such, elites that control these industries stand to profit from policies that prioritize continued operations over the well-being of the workers, thereby increasing workers' susceptibility to illness and pre-mature death (27, 28).

Filipino Americans have a long history of filling U.S. labor shortages in industries now deemed “essential,” such as nursing as well as within care, agricultural, and service economies (29, 30). The history of U.S. colonialization of the Philippines in the early 20th century, which followed three centuries of Spanish colonization, created systems and practices that facilitated the export of Philippine labor abroad (29, 31, 32). Under U.S. colonization, Filipinos were considered U.S. nationals that addressed economic imperatives and labor needs after the abolition of slavery and in light of exclusionary policies that prohibited immigration from Asia. According to Rodriguez (31), “the colonial migrant labor system could assure the United States of a continuous pool of cheap foreign labor,” unhampered by political challenges that workers from other countries posed (p. 4).

Before the Tydings-McDuffie Act of 1934 established the process of eventual independence and effectively halted Philippine immigration to the U.S., Filipinos in the U.S. during the early 1900s labored as agricultural, cannery, hospitality, and domestic workers, creating sizeable communities along the West Coast of the United States (31, 32). In addition, the U.S.-backed *pensionado* program, which initially facilitated Filipinos from elite class backgrounds to study and train in the U.S., later allowed for colonial education and training systems to proliferate broadly in the Philippines, such as the Americanized professional nursing education system (29). In her historical account of nurse migration from the Philippines, Choy (29) describes how U.S. colonial agendas created preconditions “that would lay the foundation for a gendered, racialized, and professional labor force prepared for export to the United States in the tens of thousands by the 1950's through the present” (p. 42). In all, these historical linkages help to contexualize present day conditions for Filipino American frontline workers.

Drawing on the lens offered by racial capitalism, the purpose of this study is to examine work-related health stressors and effects of the COVID-19 pandemic from the perspectives of Filipino American frontline workers. By learning about the specific contexts and dimensions of Filipino American frontline workers' experiences during the pandemic, this study aims to inform responsive policies to address conditions that shape broader social, economic, and health inequities.

## Materials and methods

Data for this research derive from a broader mixed-methods study that used a community-based participatory research approach, working in partnership with Filipino Migrant Center (FMC), a non-profit organization that serves and advocates for Filipino migrant workers and their families in Southern California. A dedicated team of FMC staff and volunteers took on roles as Community Researchers, trained in research ethics and procedures, to guide the research project aims and data collection processes, and to provide feedback on the preliminary interpretation of findings.

Community-based participatory research (CBPR) – with its fundamental principles of centering community-identified needs and perspectives, building on existing strengths and assets within communities, and using research to address health inequities and advance social justice – shaped the development and implementation of the broader study. CBPR is part of a broader movement toward developing decolonizing methodologies that challenge the ways in which dominant health research paradigms can exclude or exploit marginalized communities and, instead, begins with and permeates throughout its process communities' experiences and forms of knowledge (33, 34).

The intentional use of focus group research allowed for obtaining rich data and emic perspectives of the specific experiences, contexts, and dimensions of Filipino American frontline workers' health-related risks and effects of the pandemic. Given the population of interest, the group interview setting encouraged active, open discussion among participants with shared experiences working on the frontlines, while creating space to explore similarities and differences. Prior research indicates focus groups are an especially effective method for researching minoritized populations who mistrust, are unfamiliar with, or have not been invited to take part in research (35, 36).

### Recruitment and participants

Using a combination of convenience and snowball sampling, participants were invited to take part in focus groups as part of FMC's broad network of community contacts. Recruitment took place via postings on social media, email invitations, announcements at community events, and word of mouth. To be eligible, focus group participants needed to: (a) identify as Filipino or Filipinx/a/o American, (b) be 18 years or older, (c) speak Tagalog or English, (d) live in California, and (e) be currently employed as a frontline worker in the industries of either healthcare, retail, food services, caregiving, education, or janitorial services. Potential participants who were interested in taking part in focus groups were further screened, received an information sheet about the study, and provided oral consent. Research-trained FMC staff and Community Researchers served as facilitators for focus groups. All procedures were approved by the UCLA Institutional Review Board.

The research team conducted nine focus groups with a total of 35 participants from December 2021 through April 2022. To increase access and participation, four focus groups took place in-person and five online via Zoom. In-person focus groups were video and audio-recorded, and online focus groups were recorded on Zoom with automated transcription enabled. Five of the nine focus groups were conducted in Tagalog, and an online service was used to transcribe and translate focus group data in English. University and Community Research team members cleaned and verified all transcripts against video and audio recordings, including translated transcripts by team members fluent in Tagalog.

The 35 participants ranged in age from their early 20's through late 70's. Most were born outside the U.S. (91%) and female (80% female, 17% male, and < 1% genderqueer). Industries represented include healthcare (20%), caregiving (40%), education/childcare (20%), food services (8.5%), and retail (11.4%). All participants were employed in the Southern California region (see Table 1).

**Table 1:** Participant characteristics.

**Participant**	**Industry**	**Age range**	**Gender**	**Migrant generation**
Participant 1	Healthcare	35–39	Female	2nd-generation (U.S. born)
Participant 2	Healthcare	60–64	Female	1st-generation (migrant)
Participant 3	Healthcare	75–79	Female	1st-generation (migrant)
Participant 4	Healthcare	35–39	Male	1st-generation (migrant)
Participant 5	Healthcare	35–39	Female	1st-generation (migrant)
Participant 6	Healthcare	40–44	Female	1st-generation (migrant)
Participant 7	Healthcare	30–34	Female	1st-generation (migrant)
Participant 8	Caregiving	70–74	Female	1st-generation (migrant)
Participant 9	Caregiving	55–59	Female	1st-generation (migrant)
Participant 10	Caregiving	55–59	Female	1st-generation (migrant)
Participant 11	Caregiving	50–54	Female	1st-generation (migrant)
Participant 12	Caregiving	65–69	Female	1st-generation (migrant)
Participant 13	Caregiving	30–34	Male	1st-generation (migrant)
Participant 14	Caregiving	20–24	Female	1st-generation (migrant)
Participant 15	Caregiving	50–54	Male	1st-generation (migrant)
Participant 16	Caregiving	60–64	Female	1st-generation (migrant)
Participant 17	Caregiving	45–49	Female	1st-generation (migrant)
Participant 18	Caregiving	45–49	Female	1st-generation (migrant)
Participant 19	Caregiving	45–49	Female	1st-generation (migrant)
Participant 20	Caregiving	60–64	Female	1st-generation (migrant)
Participant 21	Caregiving	60–64	Female	1st-generation (migrant)
Participant 22	Education/Childcare	30–34	Female	2nd-generation (U.S. born)
Participant 23	Education/Childcare	20–24	Genderqueer	2nd-generation (U.S. born)
Participant 24	Education/Childcare	66–69	Male	1st-generation (migrant)
Participant 25	Education/Childcare	30–34	Female	2nd-generation (U.S. born)
Participant 26	Education/Childcare	25–29	Female	1st-generation (migrant)
Participant 27	Education/Childcare	25–29	Female	1st-generation (migrant)
Participant 28	Education/Childcare	55–59	Female	1st-generation (migrant)
Participant 29	Food Services	25–29	Female	1st-generation (migrant)
Participant 30	Food Services	20–24	Female	1st-generation (migrant)
Participant 31	Food Services	20–24	Male	1st-generation (migrant)
Participant 32	Retail	25–29	Male	2nd-generation (U.S. born)
Participant 33	Retail	25–29	Female	2nd-generation (U.S. born)
Participant 34	Retail	40–44	Male	1st-generation (migrant)
Participant 35	Retail	75–79	Female	1st-generation (migrant)

### Interview guide and procedure

A focus group interview guide and facilitation format were developed alongside FMC Community Researchers and informed by the study's conceptual framework, along with guidelines from Hall (35). The research team ensured cultural validity and appropriate translation and back-translation of interview questions in consultation with community members. The interview guide covered five general areas. The current study focused on questions related to the pandemic's effect on participants' work and health. Sample questions included: What is your job/occupation and what do you like most about your job? What are some common problems in your workplace? What problems emerged during the pandemic? What were some of the effects of the pandemic (such as health, housing, work) on you personally?

In order to address power differentials between participants and research team members, focus group facilitators emphasized that participants were experts in their own experiences and that the research team aimed to learn from them. On average, the duration of focus groups was 2 h. All participants received a $50 gift card as compensation for their time and participation.

### Researcher positionality

Trained as an interdisciplinary social work researcher, my grounding in ethnic studies provides a critical perspective I adopt in my research to examine power relations embedded within and historical linkages to current social and health inequities. As a second-generation Filipino American woman, my intersectional identities overlap and diverge from the experiences of participants in this study. I grew up in a neighborhood with a large concentration of low-income, working-class Filipino American families that mirrors the communities from which the study participants were drawn. However, my status as second-generation (U.S.-born) with rights afforded as a U.S. citizen departs from the migrant majority that comprise the study sample. My current status as a university professor extends class privilege that is denied to many of the participants, particularly those working precariously within the caregiving, retail, and service economies. My aim in conducting this research is to examine and elevate the health experiences of Filipino Americans, who are often obscured in aggregate data of Asian Americans.

### Analysis

Reflexive thematic analysis (TA) guided the analytic process (37, 38). The author read through the first transcript and made initial observations about the data, including thoughts, questions, and potential codes. The first phase of coding involved in a combination of line-by-line, *in vivo* coding (derived directly from participants' words and phrases) and concept coding, or analytic coding, in which larger units of data were assigned meaning based on theoretically relevant concepts (39). With each completed focus group, the author read through the entire transcript and listened to recorded focus group audio simultaneously, drawing on first-phase coding procedures to generate, consolidate, revise, and apply codes to data. Redundant and overlapping codes were consolidated and refined. Subsequently, a second phase of axial coding enabled higher levels of abstraction to create broader conceptual categories, subthemes, and themes. Throughout this process, reflexive analytic memoing took place for each transcript as well as various points throughout the analytic process. Community partner debriefing and member checking served as an additional measure to ensure rigor and trustworthiness (40).

## Results

Data were organized into three primary themes: (1) work-related stressors, tensions, and trauma, (2) anti-Asian racism and intersections with age- and gender-based violence, and (3) working while ill and distressed. Table 2 provides a map of the study themes and subthemes.

**Table 2:** Thematic map.

**Theme**	**Sub-theme**	**Sample quote**
Work-related stressors, tensions, and trauma	Intensified workloads and additional responsibilities	You made me become something that wasn't even supposed to happen - a teacher, a therapist, a caregiver, a [recreation] aide, everything under the sun, and a janitor, because I had to clean all the toys every day that the kids touch, we had to wash every day
	Inadequate health and safety protections	So, when the supply was short I think they were trying to have backup so we don't run out so. People on the floor were restricted - okay one mask, one gown and things like that - but they were hoarding them from whatever donations that they can get. Because when it all started, a lot of organizations knew that there was shortage of PPE, so a lot started to donate whatever they had, and I'm speaking only for my hospital. I know our hospital had hoarded those supplies. They didn't release it right away to the floor because there were trying to ration as much as they can, but then. You know, if you're on the floor and you're reusing the same thing over and over and then COVID is everywhere, it doesn't really make you confident or feel safe working, putting that mask on over and over
	Critical perspectives of employers	At the end of the day, it's really the workers that are affected because it's not like we got, like, hazard pay, or it's not like we're getting any bonus if we meet this goal. So I think that's a very big problem, and you know the management, they don't really appreciate you at the end of the day, and they expect you to even [work] overtime
	Workplace trauma	When people crash like that, when they do code blue, that's not the only patient. After patient passes away, you still have to take care of your other patients, so you have to put on another face and work like nothing happened. Carry all this emotional baggage, this, you know, just go through your 12 hours.
Anti-Asian racism and intersections with age- and gender-based violence	Elders as targets of racist violence	The people in the car said, “Asian.” I immediately thought that there was hate for Asians going on. The other one came down from the car. Chasing me and throwing things at me. I was thankful that somebody came and blocked them, they even brought me to where I was working. I did not do anything at that time because I was trembling in fear
	Healthcare workers racialized and viewed as “disease carriers”	Because of wearing scrubs… we're spreading the virus. And it doesn't help that you're Asian, you're wearing scrubs, so aside from that Asian violence, it's also the, you know, you're being discriminated because they think you're carrying the virus
	Gendered racism	We would get a lot of like slurs from customers, and I'd even say some sexual harassment. The women in the drive-through, like, there would be some male customers who would really just ask for your number, even just ask, “Would you f— me after work?” Like, straight up say that. And you know, you would report it. The management is like, write a report about it, but sometimes, because it's so busy, like, we're just forced to like brush it aside
Working while ill and distressed	Mental health issues: Anxiety, depression, and burnout	As for me, my anxiety is like the ones mentioned earlier, I just cry when I go to work… I was also really burnt out, as if I couldn't take it anymore. I'm not the type who complains at work, even though– I'm not the type who is always absent from work, but at that time, it really felt like I don't want to go into work anymore
	Physical health issues	I think there are points where you kind of see yourself… when things are quiet you're just kind of like, Oh my God, I am not okay. You know, and then you start not sleeping, and then you start eating weird, and you start eating what you can… like the health stuff goes up and down

### Work-related stressors, tensions, and trauma

A core pattern that cut across all participants involved increased workloads due to understaffing, attributed to workers out sick with COVID or resigning due to overwhelming stress and lack of organizational support. For example, within hospitals specifically, nurses on frontlines assumed the burden of direct care to ill patients battling the virus. As lower-status workers within the organizational hierarchy of the healthcare system, nurses took on the brunt of emotional labor, overwhelming hours on-call, and regularly witnessed death.

#### Intensified workloads and additional responsibilities

A core pattern that cut across all participants involved increased workloads due to understaffing, attributed to workers out sick with COVID or resigning due to overwhelming stress and lack of organizational support. For example, within hospitals specifically, nurses on frontlines assumed the burden of direct care to ill patients battling the virus. As lower-status workers within the organizational hierarchy of the healthcare system, nurses took on the brunt of emotional labor, overwhelming hours on-call, and regularly witnessed death.

I think during the surge, I felt the support from my coworkers. We helped each other out because we didn't really have doctors around. When the patient's about to die or when they're about to code, that's when the ER doctor comes, but then other than that it's just us. (Nurse, female, age 30–34).

As policies around COVID-19-related health and safety standards frequently changed, workers took on extra duties beyond their immediate scope of work:

[My employer] made me become something that wasn't even supposed to happen - a teacher, a therapist, a caregiver, a [recreation] aide, everything under the sun, and a janitor, because I had to clean all the toys every day that the kids touch, we had to wash every day. (Child care worker, female, age 30–34)

An additional responsibility that workers took on involved policing mask-wearing. Participants across industries reported challenges having to enforce mask-wearing with patients, students, customers, and colleagues. They acknowledged that constantly changing and conflicting mask-mandates contributed to widespread confusion, and interacting with people who refused to wear them contributed to awkwardness and worries about potential conflict or getting infected with COVID-19, particularly in light of uneven power dynamics in the workplace.

There was the student that I was working with and I was dealing with all day. And then, you know, he came to school, [we learned] he was positive [for COVID-19]. So that was really hard. We just live 1 day at a time, we don't know if this person is positive or not, that's the hardest part. We try to tell [students] to really keep their masks on but some of them are saying no, they are allergic to the mask, so that's why they remove it. (Paraeducator, female, age 55–59)

I haven't had the courage to tell him, You've got to step up your safety. Because he's the teacher and I'm the aid to his students. So it's like, I feel like it's not in my place. So that's been kind of weird, trying to like navigate when to speak up. (Paraeducator, female, age 30–34)

Workers also took on the burden of emotional labor during this period of prolonged uncertainty. Serving on the frontlines made workers vulnerable to the public's expressed anger and frustration to systems beyond workers' control, such as supply-chain shortages. As a result, workers were often the target of mistreatment and outsized demands and expectations.

Just the underlying amount of stress on top of just regular stress of being in retail. People asking, “Why is this not in stock?” Like, do you want me to call China and tell people to manufacturer a computer real quick? Like, I can't do that. I don't control supply chains, I don't control these things. (Retail worker, female, age 26–29)

Despite additional responsibilities and occupational hazards associated with COVID-19, most workers, particularly non-unionized workers, were not compensated with hazard pay or given sufficient paid sick leave. Instead, management provided staff with pizza or pastries during work breaks and displayed posters that hailed workers as “pandemic heroes.” As one participant stated, “I'm sick of being called a hero – I want to get paid like one. So it's kind of frustrating to just continue and be thrown in the fire as a frontliner.” (Child care worker, female, 30–34). Still, among precarious workers and those who were undocumented and regularly denied fair compensation, such recognition or trivial fringe benefits were non-existent.

#### Inadequate health and safety protections

Participants described insufficient access to PPE and on-site testing across various industries and work settings, particularly during the 1st year of the pandemic. Non-healthcare workers often bought their own PPE and sanitizing products as they were not consistently provided by many employers, placing a heavier financial toll on low-wage and precarious workers. Lower wage workers also took time outside of work to visit free, public COVID-19 testing sites regularly as a means to ensure they remained healthy while working.

Healthcare workers in hospitals were acutely affected by the lack of PPE given their role on the frontlines treating very sick patients. Although the shortage of PPE at the start of the pandemic was ubiquitous, hospital workers expressed frustration with supervisors and hospital administration's lack of transparency regarding the availability and process of rationing supplies. Tensions with leadership, coupled with the struggle to manage the influx of patients, contributed to a sense of demoralization.

When the supply was short I think [hospital administrators] were trying to have backup so we don't run out… They didn't release it right away to the floor because there were trying to ration as much as they can, but then. You know, if you're on the floor and you're reusing the same thing over and over and then COVID is everywhere, it doesn't really make you confident or feel safe working, putting that mask on over and over. (Nurse, female, age 40–44)

Across all industries, workers described ongoing worries and vigilance regarding exposure to the virus at work in light of concerns over employment security and economic pressure to provide for their families, as well as to protecting elderly and vulnerable family members from infection.

#### Critical perspectives of employers

The inability of employers to prioritize the health and safety of their workers (via appropriate PPE, regular testing, and work environment safeguards) in light of uncertain and overwhelming working conditions underlined existing power inequalities within organizations. Participants critiqued the policies of management, which they perceived as prioritizing sustained operations over workers' well-being.

At the end of the day, it's really the workers that are affected because it's not like we got hazard pay, or it's not like we're getting any bonus if we meet this goal. So I think that's a very big problem, and you know the management, they don't really appreciate you at the end of the day, and they expect you to even [work] overtime. (Food service worker, female, 25–29)

I think during COVID, we were always complaining like, Why are we still open? I remember being slightly annoyed, and being scared when I had to come into work. I just would see people buy unnecessary things. And I'm like, you should be home, I should be home, what are we doing, why are you buying these video games? (Retail worker, female, age 25–29).

In addition, workers discussed how employers created policies that, in some cases, appeared uninformed and detached from frontline working conditions.

So [administrators] are creating all these policies and all these things that some of them seem unrealistic, some of them seem ridiculous, some of them seem okay, we can do that. But how do they know unless they're in there with us? So all these people on the higher ups are making these policies and these decisions without the voices of people who are actually doing the work. All the childcare people, all of the custodians, everyone just didn't know what was going on, but they shoved us back in. (Child care provider, female, 30–34)

#### Workplace trauma

Healthcare workers, particularly nurses, described the intensity and pressures of working with the increasing volume of COVID-19 patients in the early phase of the pandemic before vaccines were available. As one participant who worked as an ICU nurse for several years stated: “I have never experienced this much death and dying” (Nurse, male, age 35–39). Participants commented about the volume of patients who were admitted to the hospital whose condition deteriorated quickly and sometimes unpredictably, and the emotional labor involved in the provision of care to patients infected with COVID-19. Despite hearing about exceptions with colleagues at other facilities, there was widespread perception among healthcare workers that the psychological effects of this pervasive trauma were not acknowledged among employers, nor opportunities provided to process these experiences.

When people crash like that, when they do code blue, that's not the only patient. After the patient passes away, you still have to take care of your other patients, so you have to put on another face and work like nothing happened. Carrying all this emotional baggage, this, you know, just go through your 12 h. (Nurse, female, age 40–44)

Though healthcare workers encountered workplace trauma more regularly, workers in other essential industries were impacted by traumatic events. One participant employed in retail described how a coworker had passed out in the store and was taken to the hospital, where the coworker died a few days later. Store employees were told the cause of death was a “medical anomaly” unrelated to COVID-19, and after 1 day of the store closing, it reopened to “business as usual.”

A part of me always thinks, Did we contribute to that? Like as a company, or the store? And I think that's also like why so many people got a wake-up call and left because they're like, it's traumatizing. (Retail worker, female, age 25–29)

### Anti-Asian racism and intersections with age- and gender-based violence

Participants reported direct experiences of racialized violence, including forms of verbal harassment, physical assault, avoidance or shunning, and comments that contributed to a hostile work environment. In addition, participants were aware of other immediate family or community members who experienced some form of anti-Asian racism. Vulnerable subgroups included older Filipino migrant women (subtheme 3.2.a) and workers employed in health-related fields, such as nursing and in-home caregiving, often viewed as “disease carriers” (subtheme 3.2.b). This theme also includes examples of gendered racism in the form of sexual harassment at work (subtheme 3.2.c).

#### Elders as targets of racial violence

High-profile media stories that have featured elder Asian American victims have caused widespread concerns about safety, particularly for the most vulnerable in these communities. Older participants reported increased vigilance when going out, and many were afraid to leave their homes unless it was deemed absolutely necessary. Participants who were older migrant women reported discriminatory and violent encounters commuting to and from work. In one instance, a participant was physically assaulted during a stop to the grocery store before work. She explained that a car with two passengers approached her, in which one person threw a glass bottle at her and another got out of the car to run after her; fortunately, a bystander intervened:

The people in the car said, “Asian.” I immediately thought that there was hate for Asians going on. The other one came down from the car. Chasing me and throwing things at me. I was thankful that somebody came and blocked them, they even brought me to where I was working. I did not do anything at that time because I was trembling in fear. (Caregiver, female, age 60–64)

Older women participants who relied on public transportation commonly experienced harassment. As one participant shared:

I also had an experience near the bus stop with a man, he said, “Are you Chinese or Korean?” I didn't mind him. He continued, “Why you're still here? Why you don't go back to your country?” I didn't reply and I just ran to the other bus stop.” (Caregiver, female, age 45–49)

#### Health workers racialized and viewed as “disease carriers”

Healthcare workers specifically mentioned that they no longer want to be seen in public wearing scrubs. Two nurses explained how being healthcare workers of Asian descent made them feel like potential targets of violence, and these fears were amplified by knowing other healthcare workers directly who experienced harassment while out in the community.

(Nurse, female, age 35–39): Because of wearing scrubs… we're spreading the virus. And it doesn't help that you're Asian, you're wearing scrubs, So aside from that Asian violence, it's also the, you know, you're being discriminated because they think you're carrying the virus.

(Nurse, female, age 40–44): I think [colleague] got attacked right?

(Nurse, female, age 35–39): Was not physically attacked but verbally. She was told at the store, she needs to “go home” [to the country she came from], because she may have the virus, because she was wearing scrubs.

A home health worker discussed her experience riding the bus with a fellow worker wearing scrubs, indicated they felt shunned by others who seemed scared of them:

Yes, especially when you have a scrub suit on, they know you work in the hospital. One time [a friend] and I, we got on the bus, they saw us in a scrub suit and asked, “Are you working in the hospital?” I said, “We're doing one-on-one private duty.” [Her friend] said, “Oh, Ate [older sister/female friend], let's not go out wearing a scrub suit.” We were told not to wear scrub suits when going out. If they see you walking, they automatically think, Where did she come from? Maybe she's infected. Especially if you work in nursing homes. (Caregiver, female, age 45–49)

These experiences instilled a sense of fear in participants, constraining their desire to be out in public and their ability to engage in everyday activities outside of their home or workplace.

#### Gendered racism

In addition to discriminatory and violent acts, women participants reported sexual harassment on the job, particularly in occupational settings wherein the majority of workers were Filipino women. Examples of this form of workplace harassment included inappropriate, disturbing comments as well as offensive, unwanted sexual advances. Participants expressed that the broader work climate discouraged the practice of addressing these forms of harassment.

We would get a lot of like slurs from customers, and I'd even say some sexual harassment. The women in the drive-through, like, there would be some male customers who would really just ask for your number, even just ask, “Would you f— me after work?” Like, straight up say that. And you know, you would report it. The management is like, write a report about it, but sometimes, because it's so busy, we're just forced to like brush it aside. (Food service worker, female, age 25–29)

### Working while ill and distressed

Pandemic stressors contributed to severe symptoms of mental and physical health problems. Anxiety, depression, and burnout were common psychological challenges that workers faced across all industries represented among participants (subtheme 3.3.a). In particular, participants' concerns about falling ill and not being able to work, as well as virus transmission fears, contributed to anxiety about threatening their family's health and economic stability. In light of workplace trauma, some participants were directly affected by death on the job, while others were haunted by the prospect of their own deaths as a result of their work. Many participants expressed pressure to continue working despite physical illness and other health ailments (subtheme 3.3.b).

#### Mental health issues

Participants described heightened symptoms of anxiety, depression, and burnout as a result of working conditions during the pandemic. Anxiety, in particular, was a dominant psychological response. Many participants expressed relentless worry related to concerns about contracting COVID-19, which had the potential to hinder their ability to continue working and provide financially for their families. As one participant expressed: “I am really scared since it is just my son and me. What if something happens to me? How about my child? He doesn't work. Student. That's what I'm most worried about. It's not easy being a mom, and it's even more difficult if I get sick, or maybe die – then what?” (Caregiver, female, age 60–64).

Given already precarious working conditions, many caregivers and home health workers had economic concerns about losing their jobs or missing work due to illness. These participants were all migrants, many of whom were employed on a contract basis or, if undocumented, paid “under the table” and are therefore ineligible for employment benefits such as sick leave, a requirement in the state of California. As one participant expressed, “I was so scared to lose my job - where I'd sleep? Or if I'll become homeless here?” (Caregiver, female, age 50–54). Still, many continued to work despite occupational hazards. The lack of formal benefits and employment protections fueled a sense of anxiety. One participant contrasted her circumstances with those of her patient in the event each was affected by life-threatening illness:

He is rich. If he got sick, he said, if he was in a vegetative state, he would just want to die. How about me? I have no money. If I get sick, where will I be picked up? There is no assurance that when you will be hospitalized, you will be treated there the same way as he was treated or if you will recover or if you will die straight away. Those are my worries. (Caregiver, female, age 45–49)

Participants expressed concerns about the potential to contract COVID-19 and spread the virus to vulnerable family members, particularly elderly and immunocompromised members. These fears were complicated by having multiple family members across generations employed within essential industries. Efforts to maintain physical distance affected family bonds and time usually spent together. A caregiver reported, “You can't see your family… Of course, you are afraid that they might get infected” (Caregiver, female, age 60–64). In addition, many participants felt an overwhelming sense of obligation to protect family members' health, and were burdened with guilt from engaging in high-exposure activities at work as well as the prospect of infecting loved ones at home.

Prior to the widespread availability of vaccines, frontline workers navigated uncertainty with regards to how the virus spread, and were additionally confronted with a lack of appropriate PPE to protect themselves from contracting the virus. As trauma and death became prevalent for hospital workers, many nurses expressed constant anxiety and worry about their ability to cope and keep up with the pressure of keeping patients alive:

During the COVID surge I had anxiety before going to work because you'd expect the worst. You know, like, I asked myself: oh my God… how many patients are going to die today? (Nurse, female, age 30–34).

A number of participants described having active symptoms of depression, anxiety, panic, and burnout while working on the job. For example, in-home caregivers and health workers who stayed with their patients during phases of lockdown were especially isolated in patients' homes, fueling depression and anxiety. For retail and service workers, having to respond the barrage of angry, demanding customers was particularly distressing. It was common for participants to minimize their symptoms or push past their emotions in order to continue working.

Sometimes I cry. My patient was like, “Are you okay?” You are crying because of your depression. Why is it like this? Life is so hard… so I just cry in the corner. (Caregiver, female, age 50–54)

As for me, my anxiety is like the ones mentioned earlier, I just cry when I go to work… I was also really burnt out, as if I couldn't take it anymore. I'm not the type who complains at work. I'm not the type who is always absent from work, but at that time, it really felt like I don't want to go into work anymore. (Caregiver, female, age 45–49)

I've had customers kind of berate me a little bit sometimes and sort of give that verbal pressure on the things that they want. There were moments, where it was so severe where I ended up, well at the time, it kind of felt like I was having a heart attack… my heart was pounding and everything. But looking back on it now, I feel like it might have been like minor cases of a panic attack. (Retail worker, male, age 25–29)

#### Physical health issues

In addition to mental health problems caused by work- and pandemic-related stress, participants faced numerous health issues. Participants reported a range of negative physical health effects of work-related stress, including poor sleep and eating habits, weight gain, pain, and migraines. Additionally, participants who were immunocompromised or had existing comorbidities described being especially hypervigilant about taking measures to protect their physical health.

I think there are points where you kind of see yourself… when things are quiet you're just kind of like, Oh my God, I am not okay. You know, and then you start not sleeping, and then you start eating weird, and you start eating what you can… like the health stuff goes up and down. (Paraeducator, female, age 30–34)

As for wellness, I just have a bit of a migraine. I'm crying so much because my vein in my head here is swollen and then my migraine is already hurting. I'll just close my eyes and then relax, inhale, exhale, that's it. Then I'll just take a shower, wash my face so they won't notice that I'm crying. (Caregiver, female, age 45–49)

Some participants contracted COVID-19, or knew of coworkers who continued working despite testing positive for the virus, and continued to work out of economic necessity.

It happened during December when I was working with a patient… I didn't think that my patient had COVID, and she tested positive. After that, she infected me with COVID. We were both quarantined for 20 days. I still work even though I got sick and then that was the worst thing that happened in my life because I didn't know what would happen to me. (Caregiver, female, 60–64)

And most of my staff came back positive still just because they wanted to go back to work, because they needed money you know it's like, that's their paycheck so it's a struggle to be like, Oh just come back when you're negative. They're like, I don't have money, you know, like, I have to come back. So they just like mask up. But now that the [indoor] mask [mandate] is gone, you know, like we just all kind of just do our best. (Child care worker, female, age 30–34)

## Discussion

The purpose of this study was to examine the work-related health stressors and consequences of the COVID-19 pandemic for Filipino American frontline workers through the lens of racial capitalism. The first two themes highlight the economic and occupational conditions as well as experiences of racism that shaped the nature and severity of stressors Filipino American frontline workers faced. The third theme captures the physical and psychological toll these pandemic stressors took on workers' well-being.

The first theme presents emergent as well as existing occupational stressors that became magnified during the pandemic. Although the increase in employee workload occurred across various industries, occupational and organization hierarchies exposed lower-status frontline workers (e.g., nurses, para educators, in-home caregivers) to greater health risks and stressors compared to higher-status workers (e.g., doctors, teachers, remote workers). This finding aligns with the perspective that workers deemed “essential” during times of crisis, and whose health and safety are most “at risk,” are often those whose labor is undervalued (41). Workers took on additional tasks well-beyond their traditional scope of work, often with minimal organizational or systemic support and a lack of appropriate resources to do their jobs safely. Along these lines, many workers felt inadequately protected from COVID-19 as many employers failed to provide appropriate PPE and testing options for frontline workers. These factors profoundly affected nurses, for example, who often shoulder the majority of bedside care for acutely ill patients. In response, workers within formal organizations and industries (e.g., healthcare, education) grew critical of government, industry, and organizational leaders who created the policies that dictated procedures workers followed but were disconnected from actual conditions on the frontlines that affected their workload, health, and safety.

Along lines of occupational hierarchy, caregivers within informal employment arrangements were provided with even fewer health and safety protections (e.g., sick leave), and many paid out-of-pocket to purchase their own PPE and took time during non-work hours to get tested. With the exception of unionized workers, most workers were not financially compensated, via hazard pay, nor informed of their rights to sick leave or other benefits, despite additional work and expanded responsibilities at work (28).

A chilling consequence of COVID was greater exposure to workplace trauma. The study findings provide further empirical data to affirm journalistic accounts of COVID-related trauma and its effects on healthcare workers (42). For those in healthcare and other industries, workplace trauma and employer's handling of it encouraged some workers to resign.

The surge in pandemic-related anti-Asian hate incidents across the U.S. has caused fear and distress for many Asian Americans. For Filipino American frontline workers, their inability to work from home inherently exposed them to greater risk of discriminatory encounters both on the job as well as commuting to and from work. The second theme presents specific accounts of racialized discrimination and violence Filipino American frontline workers encountered on and off the job. Participants described vivid encounters of physical assault, verbal harassment, and being shunned. These findings are consistent with data from Stop AAPI Hate National Report (12) that describe verbal harassment, physical assault, and avoidance or shunning as the most common anti-Asian hate incidents. Racism targeting Filipino American healthcare workers further underscores their racialization as “disease carriers,” tied to a longer history of Asian Americans as “yellow peril” (29, 43).

Notably, the participants who reported experiences of physical assault in our sample were older migrant women, and were often targeted while in transit to their place of employment, including several instances that occurred while taking public transportation. Existing data also shows that Asian American women are more likely to report harassment in public (12), with media reports highlighting the vulnerability of older Asian victims (44). Though Wu et al. (45) found that U.S-born Asian Americans were more likely to report experiences of racial discrimination compared to Asian immigrants, our participatory study provided much-needed qualitative evidence to illustrate some of the effects on populations underrepresented in research, including older Asian migrants.

The third theme presents a range of mental and physical health problems linked to pandemic-related stressors. Anxiety, depression, panic, and burnout were common psychological struggles. In particular, an overwhelming and constant sense of anxiety plagued participants, who worried about the ability to continue working and providing for family as well as protecting family members' from contracting the COVID-19. The prospect of falling ill was not merely a threat to their health as individuals, but endangered the economic stability and well-being of their families. The anxiety was compounded for those with multiple family members who were part of the essential workforce. The obligation to protect the family's health was also accompanied by feelings of guilt tied to the occupational hazards they assumed in their line of work that put them and their family in harm's way. The anxiety of contracting COVID-19 was exacerbated for precarious workers, particularly those in exploitative arrangements and who lacked access to healthcare, employment benefits, and other protections. The stress of trauma exposure also contributed to feelings of anxiety and a sense of foreboding. These findings provide qualitative detail and nuance to results from other studies that have explored the psychological toll of working during the pandemic (46, 47).

Furthermore, many workers dealt with their psychological symptoms while on the job, and the ongoing stress compromised workers' ability to cope, which was further eroded by their sense of isolation. Prior research has shown avoidance as a coping strategy among Filipino Americans mediated the link between racism-based stress and negative psychosocial outcomes (48). Applied to Filipino American frontline workers, the desire to avoid negative emotions allows for the ability to continue working yet the cumulative stress can contribute to adverse psychological health. Moreover, the stress of frontline work during the pandemic, combined with lack of access to health-promoting activities, led to a spiraling of engaging in unhealthy behaviors (e.g., poor diet and sleep) that deteriorated one's physical health. Other studies have shown this pattern among the general population (49, 50).

### Implications

The study findings inform a number of recommendations for policy and research. First, frontline workers need expanded protections and benefits, such as equitable access to appropriate PPE and COVID-19 vaccines as well as health insurance and paid sick leave, in order to ensure worker health and safety. Avenues for pursuing these protections and benefits include (a) increasing the state's capacity to enforce laws that ensure employer accountability and minimize exploitation, and (b) greater worker representation through unions. Second, the pervasiveness of racialized hate and discrimination calls for multi-pronged violence prevention efforts, which include policies, interventions, and education around public safety, particularly mindful of the needs of women, migrants, and the elderly. Third, given overwhelming distress and psychological problems caused by the pandemic, increased access to mental health care that is culturally and linguistically accessible remains a priority. Finally, the study's focus on Filipino American frontline workers underscored the need to disaggregate data of Asian Americans by ethnic group in order to provide clarity and nuance in understanding disparities within the broader Asian American population.

### Limitations

Although the qualitative design allowed for an in-depth exploration of the experiences and contexts that shaped Filipino American frontline workers' health-related risks and effects of the pandemic, the findings cannot be generalized broadly to all Filipino American frontline workers. The majority of participants were female, potentially omitting gendered experiences of male and non-binary or transgender workers. Future research should strive to reflect more equitable gender representation. In addition, reliance on convenience and snowball sampling further limited comparisons with Filipino American frontline workers beyond the migrant generation. An analysis exploring whether there are differences between migrant- and U.S.-born workers merits further investigation. Finally, though the analysis describes some distinctions between workers' conditions by industry, an analysis that accounts for occupational and industry distinctions could inform more specific guidelines to effect change in particular industries and labor contexts.

## Conclusion

Despite discourse that portrays frontline workers as “pandemic heroes,” people of color, migrants, and women are disproportionately represented across this segment of the labor force, with many facing prior social and economic disadvantages that were exacerbated with the onset of the COVID-19 pandemic. The framework of racial capitalism is useful for understanding how the pandemic shaped experiences of racialized and class-based oppression for Filipino American frontline workers. The current study findings can inform interventions and policies to improve health, work environments, and labor conditions in order to support this population and others that are disproportionately affected by current and future global crises.

## Data availability statement

The datasets presented in this article are not readily available because use of data must be approved by the study's community partner. Requests to access the datasets should be directed to Cindy.Sangalang@luskin.ucla.edu.

## Ethics statement

The studies involving human participants were reviewed and approved by UCLA Office of the Human Research Protection Program (OHRPP). The Ethics Committee waived the requirement of written informed consent for participation.

## Author contributions

CS conceptualized the study (along with community partners), led the data collection (along with community partners), analyzed the data, and drafted the manuscript.

## Funding

The UCLA Asian American Studies Center and the California Asian American and Pacific Islander Legislative Caucus provided funding for this study.

## Conflict of interest

The author declares that the research was conducted in the absence of any commercial or financial relationships that could be construed as a potential conflict of interest.

## Publisher's note

All claims expressed in this article are solely those of the authors and do not necessarily represent those of their affiliated organizations, or those of the publisher, the editors and the reviewers. Any product that may be evaluated in this article, or claim that may be made by its manufacturer, is not guaranteed or endorsed by the publisher.
